# Fracture Resistance Biomechanisms of Walnut Shell with High‐Strength and Toughening

**DOI:** 10.1002/advs.202303238

**Published:** 2023-07-30

**Authors:** Lizhen Wang, Peng Xu, Huan Yin, Yanxian Yue, Wei Kang, Jinglong Liu, Yubo Fan

**Affiliations:** ^1^ Key Laboratory of Biomechanics and Mechanobiology (Beihang University), Ministry of Education Beijing Advanced Innovation Center for Biomedical Engineering School of Biological Science and Medical Engineering School of Engineering Medicine Beihang University Beijing 100083 China

**Keywords:** heterogeneous material, impact resistance, lightweight, macro/micro‐structures, mass distribution, sclereids, walnut shell

## Abstract

Walnut shell is lightweight material with high‐strength and toughening characteristics, but it is different from other nut shells’ microstructure with two or three short sclerotic cell layers and long bundle fibers. It is essential to explore the fracture resistance biomechanism of lightweight walnut shell and how to prevent damage of bionic structure. In this study, it is found that the asymmetric mass center and geometric center dissipated impact energy to the whole shell without loading concentration in the loading area. Diaphragma juglandis is a special structure improved walnut shell's toughening. The S‐shape gradient porosity/elastic modulus distribution combined with pits on single auxetic sclerotic cells requires higher energy to crack expansion, then decreases its fracture behavior. These fantastic findings inspire to design fracture resistance devices including helmets, armor, automobile anti‐collision beams, and re‐entry capsule in spacecraft.

## Graphical abstract

Walnut shell is lightweight material with high‐strength and toughening characteristics. Multi‐scale mechanisms are employed to enhance its protective performance during impact, including: asymmetrical mass distribution that alters shell's dynamic behavior; diaphragma juglandis that enhances global stiffness and prevents cracking; S‐shaped gradient shell that improves cushioning; auxetic sclereid and pits on cellwall that increase energy consumption during deformation and fracture.

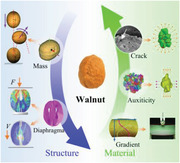

## 1 Introduction

Organisms in nature need to withstand impact loadings due to processes such as predation, combat and defense. Through long evolution, many species have developed their own unique protective mechanisms,^[^ ^1^ ^]^ which humans lack. When subjected to extreme loads, vulnerable parts of the human body, such as the head,^[^ ^2^ ^]^ neck,^[^ ^3^ ^]^ spine,^[^ ^4^ ^]^ and eyes,^[^ ^5^ ^]^ are often susceptible to injury. For plants, the use of a seed coat with high protective properties to protect seeds is a common protective strategy. Nuts have hard shell that protect seeds from broken caused by falling after‐ripening or migrations, and also resist predators’ biting with sharp teeth, which attracted attentions in field of materials and biologicals etc.^[^ ^6^ ^]^ Mechanical properties of most nuts shell were different from normal wood materials,^[^ ^6^, ^7^ ^]^ Nuts shell with similar mechanical properties had different broken resistance behaviors.^[^ ^6b,d,e^ ^]^ For example, Young's modulus of macadamia shell and walnut shell were close, but they had different toughening mechanisms.^[^ ^6e^ ^]^ Macadamia shell's microstructure was layer by layer, and varied cell morphology occurred in different layer.^[^ ^8^ ^]^ Its outer and inner layers were non‐sclerenchyma, and middle layer was the sclerenchyma composed of two short sclerotic cell layers and one long fiber bundle layer.^[^ ^9^ ^]^ When the crack formed in the inner layer, it was transferred from the sclerotic cells to the long fiber bundle in the middle layer, which would promote deflect and propagate along fiber's direction, which required higher external energy and then improve nuts shell's toughness.^[^ ^8^, ^9^ ^]^ But walnut shell had no reinforced fibers which was different with macadamia completely.^[^ ^7^, ^10^ ^]^ Indeed, the fracture strength of nutshell was related not only to the material and structure, but also the size according to the fracture mechanics theory,^[^ ^11^ ^]^ smaller size and thicker shell lead to higher axial stiffness and hardness.^[^ ^7^, ^11^ ^]^ Because there is relatively less storage volume of strain energy which means higher strain energy density is required to feed to the advancing fracture.^[^ ^11^ ^]^ Compared with macadamia shell, walnut shell has larger size, smaller relative density and thickness but its strength is larger than macadamia.^[^ ^7b^ ^]^ We guess that walnut shell should have some special structures or characteristics that guarantee good performance to resist broken as a lightweight and toughening object.

In this study, kinematics and geometry characteristics including mass center (MC) and geometric center (GC) of walnut (Video S1, Supporting Information), microstructure and mechanical behavior of walnut shell (Videos S1–S3, Supporting Information) were analyzed comprehensively. Understanding of walnut shell's broken resistant biomechanism above mentioned would inspire us to innovate protective materials or devices with excellent resistance fracture, lower mass and higher strength in the innovative design of human protection devices including helmets,^[^ ^3^ ^]^ armor, automobile impact resistant beams, re‐entry capsules in spacecraft etc.

## 2 Results

### 2.1 Kinematics and Kinetics Induced by Geometry Characteristics of Walnut

**Figure 1:**
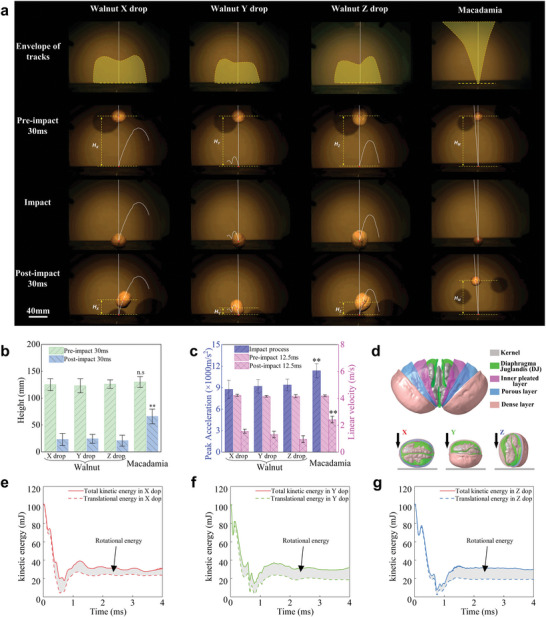
Kinematics and kinetics of walnut in dropping. a) Tracks of walnut (*n* = 20) and macadamia (*n* = 14) in dropping (Figure S1d, Video S1, Supporting Information); b) Rebound height of walnut (*n* = 20) and macadamia (*n* = 14) in dropping; c) Peak acceleration and linear velocity of walnut (*n* = 10) and macadamia (*n* = 10) in dropping; d) FE model of walnut dropped along X, Y, Z direction, respectively; e–g) Kinetic energy with time in dropping along X, Y, Z direction. (** *p* < 0.01, n.s. no significant.).

The motion trajectories of walnut and macadamia as control after dropped on a rigid plate were recorded with high‐speed camera with 2000 Hz (**Figure** **1**a). Obvious rotation was occurred for walnut in the rebounding process but no rotation for macadamia (Figure 1a; Figure S1b and Video S1, Supporting Information). The rebound height of walnut was significantly lower than macadamia, as shown in Figure 1b and Figure S1d, Supporting Information. Walnut and macadamia were at almost the same height before impact, while there was ≈2.7–3.2 times different in height at 30 ms after impact. The peak acceleration and velocities of walnut and macadamia from one meter height to the rigid plate were measured as described in Figure 1c. The velocity of macadamia was higher than that of walnut significantly. Peak acceleration of macadamia was 24.27% higher than that of walnut. The impact process was simulated using finite element method (Figure 1d; Figure S5, Supporting Information). The mechanical energy before and after impact was equal to the kinetic energy since gravitational potential energy was ignored in less than 4 ms. The rotational energy accounted about 21.1%−41.6% of the total kinetic energy in the rebound phase. The kinetic energy of rotation generated in the contact phase of impact along X, Y, Z as shown in Figure 1e–g.

**Figure 2:**
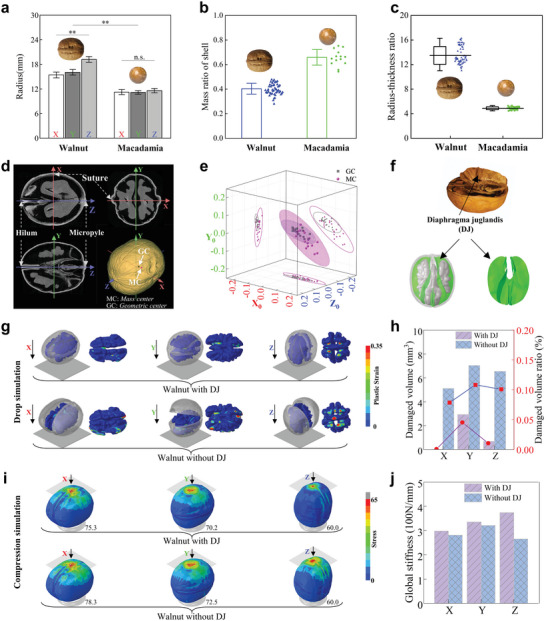
Geometry characteristics of walnut shell in macroscopic scale and role of Diaphragma juglandis (DJ). a) The radius of walnut shell (*n* = 20) and macadamia shell (*n* = 14) along X, Y, Z direction; b) Mass ratio of walnut shell (*n* = 20) and macadamia shell (*n* = 14); c) Radius‐thickness ratio of walnut shell (*n* = 20) and macadamia shell (*n* = 14); d) Micro‐CT images of walnut and coordinate axis definition; e) The normalized spatial distribution of mass center (MC) and geometric center (GC) in the 95% confidence interval (*n* = 20); f) The FE model of walnut (Video S2, Supporting Information); g) Plastic strain of walnut shell model with/without DJ in dynamic dropping; h) Damaged volume and damaged volume ratio in the walnut model with/without DJ along X, Y, X direction; i) Stress of walnut shell model with/without DJ in compression simulation; j) Global stiffness of walnut with/without DJ along X, Y, Z direction. (** *p* < 0.01, n.s. no significant).

The radius, mass ratio and radius‐thickness ratio of walnut and macadamia were compared (**Figure** **2**a–c). The shell mass was only account about 40% of total walnut mass, which was significantly smaller than that of other nuts. It was more than 65% for Macadamia (Figure 2b). The position coordinates of MC and GC was normalized and the 3D scatter points of MC and GC were projected to the three coordinate planes X_0_Z_0_, Y_0_Z_0_, and X_0_Y_0_ in the 95% confidence ellipse, respectively (Figure 2d,e). The statistical average deviation of walnut's MC and GC is about 2.93%. The average deviation of the MC along X_0_, Y_0_, and Z_0_ from the coordinate origin is about 5.43%, 0.20%, and 2.22%. The dispersion along X_0_ was the largest, then followed by Z_0_ and the minimum along Y_0_. While the average deviation of GC along X_0_, Y_0_, and Z_0_ from the coordinate origin is about 5.33%, 0.63%, and 0.87%. And the dispersion degree along the X_0_ is also the largest, then followed by Z_0_ direction, and the smallest along Y_0_. In addition, walnut has a special diaphragma juglandis (DJ) that divided into the walnut to two parts (Figure 2f). The role of DJ under the dynamic drop (Figure 2g,h) or compression loading (Figure 2i,j) was analyzed using the finite element (FE) model (Figure S5, Supporting Information) with or without DJ. High plastic strain occurred on the small contact areas of cotyledon and embryo in the model with DJ, but it occurred on a bigger area even to the front side of cotyledon and embryo for the model without DJ (Figure S6, Supporting Information). The damaged volume of walnut induced by impact was less than 0.05% as shown in Figure 2h, which means most of mechanical energy in the drop impact consumed by the walnut deformation. The suture keep closed after impact for the model with DJ, but it was fully opened in the model without DJ (Figure 2g; Figure S7, Supporting Information). The global stiffness of the model with DJ was higher than that of without DJ (Figure 2i,j). The global stiffness was ordered as X‐axis<Y‐axis<Z‐axis for the model with DJ, while it was the maximum along Y‐axis and minimum along Z‐axis for the model without DJ (Figure 2i; Figure S8, Supporting Information). The stress on the models with DJ was smaller than that of models without DJ in the area of loading (Figure 2i).

### 2.2 Heterogeneity of Walnut Shell along Outer‐Inner Direction

**Figure 3:**
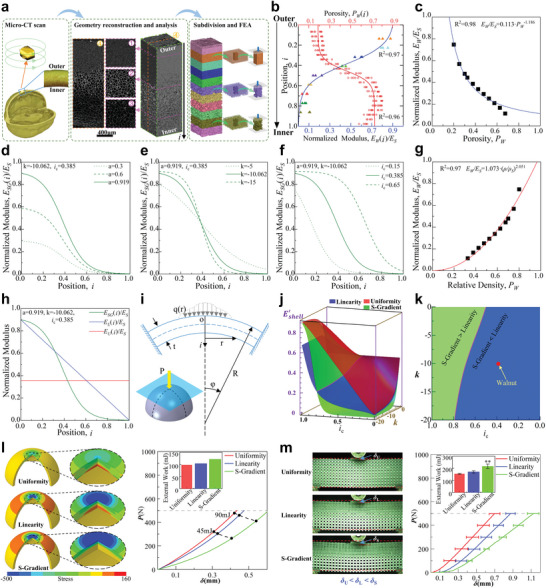
Role of non‐uniform characteristics in microscopic scale on walnut shell's fracture resistance. a) Microstructure of walnut shell based on Micro‐CT images and its subdivision (Video S1, Supporting Information); b) Porosity (*n* = 10) and normalized modulus of walnut shell along the outer‐inner direction; c) Relationship of porosity and normalized modulus; d–f) Effect of parameters *a, k, i_c_* on the function of gradient distribution; g) Relationship of relative density and normalized modulus; h) Relationship of position and normalized modulus; i) Effect of gradient porosity and modulus of walnut shell based on the simplified model; j) Relationship of three modulus distributions (i.e., uniform, S and linearity gradient) and the shell's equivalent modulus; k) Relationship between the equivalent modulus of S shape and linear gradient modulus distribution and the value of the k and *i*_c_ by solving the function. l) Simplified spherical shell model with uniform, S and linearity gradient elastic modulus and the stress, deformation and external work under the same loading condition; m) The three‐points bending testing of 3D printing samples with uniform, S and linearity gradient elastic modulus (*n* = 10) (** *p* < 0.01).

The walnut shells’ microstructure was analyzed along thickness direction (outer‐inner direction, *i*‐axis) using Micro‐CT image data. Walnut shell was heterogeneous at the local level (**Figure** **3**a). The porosity along *i*‐axis was presented as the S‐shape gradient distribution as shown in Figure 3b. The functional relationship of porosity *P_W_* (*i*) and the position *i* from outer to inner was described as follows:

(1) $$P_{W}\left(i\right)=0.7289-\frac{0.5412}{1+e^{\frac{i-0.4533}{0.0571}}},\;i\in \left[0,1\right]\;R^{2}=0.96$$

Where *i* was zero at the outermost layer, and *i* was one at the innermost layer. Elastic modulus of shell along *i*‐axis was defined as *E_W_* (*i*) and elastic modulus of singe cell walls was defined as *E_S_*. The normalized elastic modulus was defined as *E_W_* (*i*)/*E_S_*, which would avoid the effect of different base materials. *E_W_* (*i*)/*E_S_* along *i*‐axis was also found to be S‐shape gradient distribution (Figure 3b). The functional relationship of *E_W_* (*i*)/*E_S_* and the position *i* from outer to inner was described as follows:

(2) $$\frac{E_{W}\left(i\right)}{E_{S}}=\frac{0.9187}{1+e^{10.0623*\left(i-0.3854\right)}},\;i\in \left[0,1\right]\;R^{2}=0.97$$

So the functional relationship of *E_W_* (*i*)/*E_S_* and *P_W_* (*i*) was described as follows (Figure 3c):

(3) $$\frac{E_{W}\left(i\right)}{E_{S}}=0.113*P_{W}{\left(i\right)}^{-1.186},\;i\in \left[0,1\right]\;R^{2}=0.98$$

Then, the functional relationship between normalized elastic modulus *E_W_* (*i*)/*E_S_* and normalized density ρ(*i*)/ρ_*s*_ was also described as follow according to the relationship between porosity and density (Figure 3g):

(4) $$\frac{E_{W}\left(i\right)}{E_{S}}=1.073*{\left[\rho \left(i\right)/\rho_{s}\right]}^{2.051},\;i\in \left[0,1\right]\;R^{2}=0.97$$

We defined *E_SG_* (*i*)/*E_S_* was the normalized elastic modulus of walnut shell with S‐shape gradient, *E_L_* (*i*)/*E_S_* was that of linear gradient, *E_U_* (*i*)/*E_S_* was that of uniform distribution. Then the functional relationship of normalized modulus for S‐shape gradient, linear gradient and uniform elastic modulus distribution were described as follows:

(5) $$\frac{E_{SG}\left(i\right)}{E_{S}}=\frac{a}{1+e^{-k\left(i-i_{c}\right)}},\;i\in \left[0,1\right]$$

(6) $$\frac{E_{L}\left(i\right)}{E_{S}}=\frac{a}{1+e^{ki_{c}}}\left(\frac{e^{ki_{c}}-e^{k\left(i_{c}-1\right)}}{1+e^{k\left(i_{c}-1\right)}}\cdot i+1\right),\;i\in \left[0,1\right]$$

(7) $$\begin{array}{ccc} \frac{E_{U}\left(i\right)}{E_{S}} & = & \int_{0}^{1}\frac{E_{SG}\left(i\right)}{E_{S}}di=\frac{a\left(k+\ln\left(1+e^{k\left(i_{c}-1\right)}\right)-\ln\left(1+e^{ki_{c}}\right)\right)}{k},\\ & & i\in \left[0,1\right] \end{array}$$

The effect of *a, k, i*_c_ on the normalized modulus was analyzed (Figure 3d–f). *k* and *i*_c_ affect the shape of normalized modulus distribution function, while *a* affects only functional amplitude. Therefore, *a* was regarded as a constant for different modulus distributions, but *k* and *i*_c_ are independent variables.

The 2D theoretical model was established to study the effect of local elastic modulus distribution on the mechanical property of whole walnut shell (Figure 3i). According to Hertz theory,^[^ ^12^ ^]^ δ is deflection at the center point of the shell, and it is up to maximum on the center of contact area under loading *P*.

(8) P=∫q(r)dr

(9) $$\delta =\frac{a^{2}}{R}={\left(\frac{9P^{2}}{16RE^{*2}}\right)}^{\frac{1}{3}}$$

Assuming the contact surface of impact was rigid flat plate, the curvature radius and modulus of flat plate were set to ∞, respectively. The reduced modulus E*of contact zone can be simplified as follow:

(10) $$E^{*}=\frac{E_{shell}}{1-\mu_{shell}^{2}}$$

Where *E_shell_* was equivalent modulus of walnut shell along thickness, µ_*shell*_ was passion ratio of walnut shell. Based on Voigt–Reuss mixing rules,^[^ ^13^ ^]^ equivalent modulus $E_{shell}=\frac{1}{\int_{0}^{1}1/E\left(i\right)di}$. And the normalized equivalent modulus $E_{shell}^{\prime }=\frac{E_{shell}}{E_{s}}=\frac{E_{s}}{\int_{0}^{1}1/E\left(i\right)di}$. According to local elastic modulus distribution forms along thickness, the *E*(*i*) was obtained from Equations (5)–(7).

The relationship of $E_{shell}^{\prime }$, *k, i*_c_ for the three distributions was shown in Figure 3j. Purple line in Figure 3j,k were the combination of k and *i*_c_ when normalized equivalent modulus with S‐shape and linear gradient was equal. Equivalent modulus in the uniform distribution was greatest according to Figure 3j. As shown in Figure 3k, Equations (9) and (10), deflection of walnut shell with S‐shape gradient was higher when *k* and *i*_c_ located in blue areas (the equivalent modulus was S‐shape gradient<linearity), and it was opposite when *k* and *i*_c_ located in green areas. The red point located in blue area in Figure 3k presented walnut shell, which means deflection of walnut shell with S‐shape gradient modulus was highest under the same compression compared with the other two distributions. The work done by rigid body load on spherical shell in the linear elastic stage would be described:

(11) $$W_{E}=\int \;\;\;\int q\left(r\right)\mathrm{drd}\delta \approx \frac{1}{2}P\delta$$

The higher deflection δ, the higher work *W_E_* done by rigid body, which converted to deformation energy and absorbed by spherical shell. Besides, the interaction time was longer for the higher deflection δ. The buffering effect of shell with higher deflection would be more obvious based on the momentum theory. The ability of impact energy absorption in collision under the same loading was described as S‐shape gradient shell>Linearity gradient shell>Uniform shell when *i*_c_ and k were located in blue area of Figure 3k; but it was Linearity gradient shell >S‐shape gradient shell>Uniform shell when *i*_c_ and k were located in green area of Figure 3k. The loading *P* on contact area was affected by modulus distribution of walnut shell along *i*‐axis when work *W_E_* was same. According to Equations (9)–(11), the loading *P* would be S‐shape gradient<Linearity<Uniformity for the three distribution when *i*_c_ and k were in blue area of Figure 3k; but it was Linearity<S‐shape gradient<Uniformity when *i*_c_ and k were in green area of Figure 3k.

Compared to theoretical models, FE models of spherical shell with three modulus distributions are developed (Figure S9, Supporting Information). For the uniformity distribution, the maximum tensile stress occurred on the inner of shell, and the maximum compression stress on the outer. For the linearity distribution, the stress was similar with that of uniformity, and there was bigger area of tensile stress on the outer of shell. For S‐shape gradient distribution, the smallest stress on the inner and outer of shell compared with the other two distributions (Figure 3l). The deflection δ and work *W_E_* by loading were satisfied S‐shape gradient>Linearity gradient>Uniformity under the similar loading. The peak loading *P* was satisfied with S‐shape gradient < Linearity < Uniformity for the three modulus distributions when *W_E_* was same. 3D printing samples with three modulus distributions were fabricated and tested. Deflection and absorbed energy of S‐shape gradient sample was highest in the three distributions under the same loading (Figure 3m).

### 2.3 Fracture Resistance Behavior

**Figure 4:**
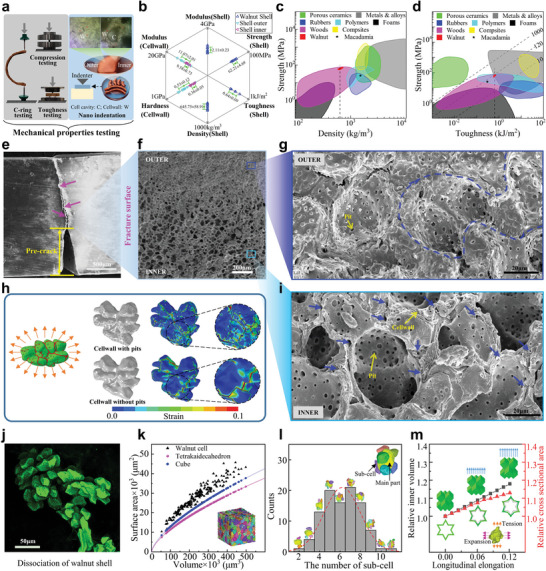
Mechanical fracture resistance behavior on microscopic scale. a) Mechanical testing of C‐ring, compression, three‐point bending and Nano‐indentation experiments (Supplementary Video 3); b) Mechanical properties of walnut shell and its single cellwall (*n* = 10); c) and d), Density‐strength and toughness‐strength of walnut shell and macadamia (data from references^[6b,6d,15]^) in Ashby plot; e) Crack propagation of walnut shell samples after bending (Video S3, Supporting Information; Purple arrow marked fracture deflection); f) SEM image near the fracture line in the interface of dense and porous areas; g) SEM image near dense area in the outer of walnut shell (fracture occurred in the middle lamellar as marked by blue dotted line); h) Crack propagation analysis based on micro FE model of single cell with/without pits (Video S3, Supporting Information); i) SEM image near porous area in the inner of walnut shell (fracture occurred by the whole cells torn and the cracks propagated along the pits of cell walls marked by blue arrows); j) Observation of dissociated single cell (Video S4, Supporting Information); k) Relationship of single cells’ volume and surface area (*n* = 210); l) The statistics of cell numbers consisted concave multicellular structure; m) The relationship of relative inner volume and longitudinal elongation for the concave multicellular auxetic structure based on numerical simulations (Video S4, Supporting Information).

Mechanical properties of walnut shell were measured by C‐ring test, compression, three points bending, nano‐indention testing (**Figure** **4**a,b,e; **Table** **1**; Figures S3 and S4, Supporting Information). Density of walnut shell was obtained as 645.75 ± 58.91 kg m^−^^3^. There is no significant difference for cellwall's modulus near the inner and outer of walnut shell and the mean value of 10 225 MPa was set as cellwall's modulus for walnut shell (Figure S4d, Supporting Information). However, the hardness of single cellwall near the outer side was about 1.48 times higher than that of the inner side (Figure S4g, Supporting Information), which resulted in higher wear resistance. So mechanical properties of walnut shell located in the border of wood materials in the Ashby strength‐density plot (Figure 4c), which means the strength of walnut shell was one of the highest in the wood materials with same density. In the Ashby strength‐toughness plot, the diagonal line represented the isoline of strength and toughness's ratio with 120 km^−1^, which means the strength of walnut shell was highest in the wood materials with same toughness (Figure 4d). There is deflection and fluctuation during the crack propagation, which means that it would resist the crack propagation inside walnut shell (Figure 4e). The middle lamella broken occurred near the outer side and cell wall was integral, but the cellwall broken occurred near the inner side (Figure 4f,g,i). The path of crack pass propagation through many pits (which are marked by blue arrows) as shown in Figure 4i and Figure S12, Supporting Information. FE model of single sclereid was developed (Figure S10a, Supporting Information). Compared with cellwall without pits, abundant micro cracks appeared on the cellwall with pits. The pits deflected and spited micro cracks, which may increase the fracture energy (Figure 4h). The micro‐structures of walnut shell's single sclereid were dissociated using confocal microscopy, which was consisted of puzzle‐shaped cell with equal diameters sub‐cell, but no special long axis direction (Figure 4j). The superficial area, volume, and the sub‐cell number of walnut shell's single cell were calculated (Figure 4k,l). The superficial area of walnut shell cell was bigger than that of cube or tetrakaidekahedron with same volume, which means walnut shell was concave multicellular structure with bigger cell superficial area. And the sub‐cell number fits the normal distribution with the average of 6.21 (Figure 4l). So single sclereid of walnut shell can be simplified as the 3D cube cross model (Figure S10b, Supporting Information), And auxetic behavior has been found during cellwall deformation as shown in Figure 4m.

**Table 1:** Summary of mechanical properties of walnut

Testing methods	Hardness [GPa]	Modulus [GPa]	Yield Strength [MPa]	Break Strength [MPa]	Maximum Plastic Strain	Toughness [kJ m^−2^]
Compression testing	——	2.11 ± 0.23	48.62 ± 3.95	62.21 ± 4.69	0.116 ± 0.042	——
C‐ring testing	——	1.77 ± 0.34	——	53.64 ± 6.48	——	——
Toughness testing	——	——	——	——	——	0.64 ± 0.09
Nano indentation	0.532 ± 0.124^a^ ^),^ ^c^ ^)^ or 0.359 ± 0.045^b^ ^),^ ^c^ ^)^	11.07 ± 2.01^a^ ^),^ ^d^ ^)^ or 9.38 ± 0.73^b^ ^),^ ^d)^	——	——	——	——

^a)^ outer surface;

^b)^ inner surface;

^c)^ significant difference (P<0.01);

^d)^ no significant difference (P<0.01).

## 3 Discussion

Walnut shell has better whole strength to protect nuts avoiding damage, which is also lightweight material with higher strength and toughness. It is a well example with higher strength and toughness simultaneously, which is very difficult to achieve the balance for structural materials.^[^ ^14^ ^]^ Walnut shell had highest strength and toughness than those of other wood materials with same density as shown in Figure 4c,d. For example, the density of macadamia shell was about 1.5 × 10^3^ kg m^−3^, and the strength was 25 MPa, the toughness was 0.3 kJ m^−2^.^[^ ^6^, ^15^ ^]^ And the density of walnut shell was 6.46 × 10^2^ kg m^−^^3^, the strength and toughness were 62.21 MPa and 0.64 kJ m^−2^, respectively. The density of walnut shell was smaller than macadamia obviously, but the strength and toughness were slightly higher than macadamia. So specific strength and specific toughness of walnut shell would be larger than those of macadamia.

We found walnut has special macro‐structure including non‐coincidence of walnut's MC and GC, DJ (Figures 1 and 2). Impact force usually passes through GC of the objects when collision, then the resultant force of gravity and impact force would produce a moment which induced its rotation. Walnut had rotary‐motion after rebounded, but macadamia was mainly translational‐motion. The velocity decreased 69.5% and 42.2% for the walnut and macadamia after collision, respectively. The rebound height of macadamia was significantly higher than that of walnut after collision, all of which means walnut had better resistance to impact.^[^ ^16^ ^]^ The rebound height of macadamia was higher, which makes it possible to have multiple collisions with repeated impacts, then causing damage to its shell or nuts. The lower rebound height of walnut indicated that the considerable energy after collision was transformed into rotational energy and less gravitational potential energy. Walnut will not rebound again and just roll along the plane, then remaining energy would be consumed by friction between shell and plane. The kinematics of walnut in collision caused by the non‐coincidence of MC and GC was one of important factors to avoid shell or nut damage.

DJ was the dry xylem septum of walnut kernel located the gap of kernel and shell, which supports nuts and avoids the motion of nuts in shell (Figure 2). It was found that higher plastic strain occurred in a small contact area of cotyledon and embryo in collision for the model with DJ, but it would expand to bigger areas for the model without DJ. Global stiffness of the model with DJ increased 9.6%, 10.9%, and 47.7% in X‐axis, Y‐axis, and Z‐axis, respectively compared the model without DJ in compression. Strengthening occurs via an increased shell thickness, spherical shape, small size, and a lack of extended sutures for macadamia.^[^ ^7b^ ^]^ It was totally different for walnut shell since it had bigger volume and thinner shell (Figure 2a–c). The reinforced structure of DJ connected the top and bottom of walnut shell, increased load transmission and decreased local deformation and stress. The suture model without DJ appeared more obvious cracking in collision. Global stiffness of the walnut shell was increased significantly by DJ especially along Z‐axis, which means DJ played a role of “safety partition.”

Macadamia has hierarchical microstructure composed of sclereid cells with equal diameter and sclerenchymatous fiber with different orientations,^[^ ^8^ ^]^ which improved its plastic deformation under loading. The disordered fibers increased toughness by dissipated more energy in the process of crack deflection.^[^ ^8^, ^17^ ^]^ We found the porosity and elastic modulus of S‐shape gradient distribution for walnut shell along the outer‐inner direction on microscopic scale (Figure 3b), which increased the strength of walnut shell. The higher tensile stress occurred on shell's inner surface for the linear gradient or uniform distribution of elastic modulus, which induced the crack initiation, even accelerated its expansion.^[^ ^18^ ^]^ But higher compression stress and tensile stress both occurred on the outer surface which would reduce the fracture risk of whole shell when we changed the linear gradient or uniform distribution of elastic modulus to S‐shape gradient (Figure 3l). S‐shape gradient elastic modulus distribution along thickness also enhances the cushioning effect of walnut shell in impact. Compared with the shell of uniform material, the gradient distribution increases the deformation during impact under same loading, thus increasing the ability to absorb the external work, and realizing the buffering effect; the gradient distribution reduces the force during impact under the same work done by the external force, realizes the buffering effect. Moreover, S‐shaped gradient distribution is better than the linear gradient distribution in a certain range (Figure 3k).

The porosity of walnut shell was also been S‐shape gradient distribution along outer‐inner direction (Figure 3b). The porosity of outer shell was smaller for its thicker cell wall and smaller cell cavity. And the porosity of inner shell was bigger for its thinner cell wall and bigger cell cavity (Figure 3a, Figure 4f). The strengthening and toughening mechanisms of outer and inner of walnut shell was also different. For the outer shell, crack occurred on the interface of cells for its thicker singe sclereid cell wall that difficult to be broken (Figure 4g). The puzzle shape of sclereids is one of the mechanisms for its high strength.^[^ ^7a,b^ ^]^ The form of interlocking between cells increases the area of fracture, thus increasing the energy required for the fracture process. This interlocking structure is important for improving the resistance of the walnut shell to crack expansion and increasing the energy absorption capacity.^[^ ^19^ ^]^ The hardness of the outer shell's cell wall was 1.48 times of the inner shell's cell wall, which improved the abrasion resistance.^[^ ^20^ ^]^ The puzzle‐shaped multicellular structure of inner shell increased the deformation space and enduring higher strain. The max plastic deformation was 0.116 according to the results of mechanical testing (Figure 4b). Normalized elastic modulus and relative density have a power relation of 2.051 as Equation (4). When the power exponent of elastic modulus and relative density was ≈2–3 for the porous materials, the deformation was occurred by the bending of cell wall.^[^ ^21^ ^]^

There were many pits on the single cell wall especially near inner area of walnut shell. The crack path in cell wall was analyzed based on FE model of single cell with/without pits (Figure 4h). It was found that crack propagation direction was changed by pits or spread into more small cracks after passing pits (Figure 4h), which was consistent with the blue arrow marked pits of crack crossing (Figure 4i and Figure S12 Supporting Information.). Pits was proved to be beneficial to crack deflection and increasing of toughness, which was similar to the toughening mechanisms of alligator gar (*Atractosteus spatula*)^[^ ^22^ ^]^ and ginkgo seed shell.^[^ ^23^ ^]^ Meanwhile, the single cell wall of walnut shell had concave auxetic features with six sub‐cells (Figure 4h,l). The volume of cell and the cross‐sectional area perpendicular to stretching direction increased when it was stretched according to the analysis of simplified model (Figure 4m; Figure S10b, Supporting Information). The concave auxetic structure of single cell required higher energy to crack propagation and expansion, which would increase the ability of cell's absorbing energy during deformation.^[^ ^24^ ^]^

## 4 Conclusion

In summary, the protective biomechanism of walnut shell was resulted from its relative position of MC and GC; unique geometry characteristics diaphragma juglandis (DJ); S‐shape gradient distribution of porosity and modulus along outer‐inner direction; concave multicellular morphology of single sclereid; and pits on the cellwall of sclereid. Walnut shell is a fantastic lightweight material, which is “soft” as a whole object, and “harder” at the special areas. The characteristics of above mentioned would be valuable to inspire design of new cushioning or energy absorption structures, and apply in the re‐entry module or flexible capsule in shock tunnel, etc.

## 5 Experimental Section

### Materials

Walnuts (*Juglans regia L*) from Xinjiang of China and macadamias (*Macadamia integrifolia Maiden & Betche*) from Yunnan of China were selected in this study. All walnut and macadamias samples were peeled, washed and dried based on the same procedure. The moisture content of the samples was measured to be about 5.8 ± 0.2wt%.

### Drop Testing

The kinematics and kinetics of walnut and macadamia dropping in three‐axis of walnut from the height of 1m to the rigid plate were analyzed (Figure S1a, Supporting Information). The high‐speed camera (i‐speed 3, ix‐cameras, UK) with 2000 Hz frequency was used to record the motion (tracks and linear velocity) of walnut and macadamia in the whole process of dropping. In addition, the load sensor (2527‐131, Instron, USA) was also set on the rigid plate with 5000 Hz frequency to record the impact force in collision (Figure S1a, Supporting Information). The greater the mass of the nuts will cause greater impact force during collision. In order to eliminate the effort brought by the difference in individual mass, the peak acceleration of nuts (the maximum impact force was divided by their mass) during collision was used in this study.

### Mass Center and Geometric Center of Walnut

The Plumb line method (PLM) is often used to measure the Mass center (MC) of plate objects. PLM was combined with image reconstruction to measure the MC and the geometric center (GC) of walnut in this study (Figure S2, Supporting Information). Walnut was freely suspended on plastic fixtures using soft lightweight string. X‐rays can easily pass through the plastic fixture without effect image quality. The fixture was placed in the Micro‐CT (skyscan1076, Bruker, Belgium) sample chamber and the scan was started after the walnut was completely stationary (about 10 min). For each sample, Micro‐CT scans were performed from two different suspension positions, respectively. Geometrical models were reconstructed in Mimics software 20.0 (Materialise Inc, Belgium) by the Image data of walnut and plumb line. The geometric models from the two scans were geometrically registered using the software Geomagic wrap 2017(3D systems, USA), and the intersection points of two plumb line extensions were calculated. According to the balance of forces, MC must be on the extend direction of the plumb line, so the intersection of two plumb line extensions is MC. GC is the shape center of the structure. All the points on the outer surface of the walnut were calculated as the geometric average (Equation (12)), then we can get the coordinates of GC.

(12) $$x_{GC}=\;\int \int \int \;x\mathrm{dV},\;y_{GC}=\;\int \int \int \;y\mathrm{dV},\;z_{GC}=\;\int \int \int \;z\mathrm{dV}$$

In order to compare the MC and GC of different walnuts, MC and GC of samples were converted to the coordinate system shown in Figure 2d. Data normalized (that is, the coordinate value of MC and GC data were respectively divided by the 3D size of each sample) was performed to eliminate the effect caused by the different sizes of different walnuts. Then the outer surface of the walnut transformed into a standard sphere of radius 1, MC and GC transformed into scatter points distributed inside that sphere.

### Material Properties of Walnut

1) Density. Ten Cuboid samples were cut from 10 walnuts, the mass of each sample was measured using an analytical balance, and the volume of each sample was obtained through the geometrical model which reconstructed by Micro‐CT (skyscan1076, Bruker, Belgium) scanning image data with voxel resolution 37.94 µm. The ratio of mass and volume of each sample was its density. 2) Compression testing. Cuboid samples (about 2 mm × 2 mm × 4 mm) were cut from the small curvature area in the middle of walnut shell, then inner and outer surfaces of the samples were polished with sandpaper to make the surface of the samples flat. Compression testing of walnut shell (*n* = 10) was conducted using a universal testing machine (E10000, Instron, USA) (Figure 4a) at a speed of 1 mm min^−1^. The slope at the beginning of stress‐strain curve was taken as the elastic modulus, the stress corresponding to 0.2% plastic strain was taken as the yield strength, the maximum stress on stress‐strain curve was break strength, the maximum plastic strain was obtained by subtraction of break strain and yield strain (Figure S3a,b, Supporting Information). Besides, ten cuboid samples (about 4 mm × 4 mm × 6 mm) were cut from the walnut kernel and polished with sandpaper. Compression testing was carried out at speed of 1 mm min^−1^, and the loading was stopped when displacement reach to 2 mm (Figure S3f,g, Supporting Information). **3)** C‐ring testing. In order to obtain reliable results, C‐ring tests of ten samples (r: the outer radius; b: width; t: thickness of walnut shell) were also processed by universal testing machine (E10000, Instron, USA) (Figure 4; Figure S3c, Supporting Information). Based on Castigliano's Theorem, the relationship between the deformation and loading in linear phase of C‐ring tests’ results was described as follows:^[^ ^25^ ^]^

(13) $$E=\frac{3\pi }{4b}{\left(2\frac{r}{t}-1\right)}^{3}\cdot K$$

*K* is slope of linear phase in load‐displacement, *E* is elastic modulus of walnut shell. The fracture usually occurred in the mid‐section of outer surface in C‐ring samples. Based on the curved beam theory, the break strength σ_b_ was described as follows:^[^ ^26^ ^]^

(14) $$\sigma_{b}=\frac{2F_{\max}\left(3r-2t\right)}{{\mathrm{bt}}^{2}}$$

F_max_ is the maximum load. 4) Toughness testing. Stress intensity factor ***K_IC_*** and the critical value ***J_IC_*** of ***J***‐integral are often used to characterize the toughness of materials. However, the width of the sample *B* is generally required to be larger than 2.5(***K***_***IC***_/σ_*s*_)^2^ in the testing of ***K_IC_***. Walnut shell was difficult to meet the size requirements of ***K_IC_*** test for its thinner shell. Therefore, toughness of walnut shell was measured based on the multiple specimen ***J***‐integral tests,^[^ ^27^ ^]^ (Figure 3a, e; Figure.S3d,e, Supporting Information). In the linear elastic range, the ***J_IC_*** and ***K_IC_*** are interchangeable:

(15) $$J_{\mathrm{IC}}=K_{\mathrm{IC}}^{2}/E$$

***J***‐integral at the crack tip is equal to:

(16) $$J=-\frac{1}{B}{\left(\frac{\partial U}{\partial a}\right)}_{\delta }$$

*a* is the length of the crack, *δ* is the displacement at the loading point, and *U* is the strain energy of the sample (equal to the work done by the external force on the sample under static load). When *δ* approaches critical value *δ*_IC_, the ***J***‐integral critical value ***J_IC_*** can be obtained. The three‐points bending was processed on the multiple different pre‐crack samples. The sample sizes were as follows: the initial crack depth *a* was about 0.5–0.75 of the width *W* of the sample, the ratio of thickness *B* to (W‐a) was 2–2.5, and the ratio of span to width L/W of the three‐points bending sample was about 4. The point at which the load begins to decline on the load‐displacement curve was taken as the critical point *δ_IC_* to determine the fracture toughness ***J_IC_***. Five sets of tests were conducted, each set had three to five sample with different pre‐crack depth *a* to obtain toughness ***J_IC_***. The crack depths of samples before and after testing were observed using metallographic microscope (LV150N, Nikon, Japan). **5)** Nano indentation. Nano indentation can characterize the mechanical properties of walnut shell cellwall. The resin was used to embed walnut shell samples, and the tested surfaces were respectively polished with 500, 1000, 5000, 20 000 grit sandpaper at low speed. Metallographic microscope (LV150N, Nikon, Japan) was used to examine the polished surface. Nanoindenter (G200, Agilent, USA) was used to perform in situ scan on testing area to obtain microscopic 3D morphologies for assessment of the roughness of testing area. The cellwall thickness of walnut shell was about 7.5 µm, and the roughness of testing area was less than 50 nm (Figure S4a, Supporting Information). Therefore, 1000 nm was set as the indentation depth, which can avoid the effect of substrate on test results. The positions of the indentation were precisely controlled by the microscope to locate them on the cellwall and away from the cell cavity. The modulus and hardness of the cellwall near the inner and outer surfaces of walnut were obtained by using the continuous stiffness mode of nanoindenter (G200, Agilent, USA). The test values of 100 to 400 nm indentation depth were selected as the final result, as the test values tended to be stable during this interval (Figure S4b‐f, Supporting Information). There is no significant difference for cellwall's modulus near the inner and outer (Figure S4d, Supporting Information), and the mean value of 10 225 MPa was set as the single cellwall's modulus (i.e., ***E_s_***) for walnut shell.

### Finite Element Analysis of Whole Walnut

The whole walnut model based on the Micro‐CT images was developed by using Mimics (Mimics Medical 21.0, Materialise, Belgium) and Geomagic wrap 2017(3D systems, USA).^[^ ^4^, ^28^ ^]^ The whole model including walnut shells, kernel and DJ (Figure 1d; Figure S5a, Table S1, Supporting Information), the mechanical properties were from the results of mechanical testing in this study (Table 1). Simulations were conducted by using LS‐DYNA software (LSTC, USA). On the microscopic scale, walnut shell is not uniform material, and its porosity and mechanical parameters show gradient changes. In this model, the walnut shell was simplified to consist of a dense layer and a porous layer. The density and elastic modulus of those two layers were obtained by calculating the average of the values in the region, according to the changes of porosity and elastic modulus along the thickness direction shown by Figure 3b. A thin layer of elements (0.2 mm) was built to connect the two parts of the shell. The strength limit of the connecting elements was set as 2.5 MPa according to the bonding strength of the suture of walnut shell.^[^ ^29^ ^]^ During simulation, when the stress in suture of walnut shell exceeds the bonding strength, connecting elements will be deleted for failure. Therefore, the cracking phenomenon of walnut shell overload can be simulated. Strain (>0.35) was selected as the damage criterion of kernel (Figure S3g, Supporting Information). The model was validated by the compression testing data (*n* = 6) of walnut. There were similar crack position and high‐stress area between simulations and experiments (Figure S5c,d, Supporting Information). The impact forces were also very close between simulations and experiments (Figure S5e, Supporting Information). The impact process of walnut with or without DJ from the height of 1m to the rigid plate was simulated and compared (Figure 1d–g and Figure 2g,h). The compression of whole walnut was also simulated and the stiffness was compared for the model of with/without DJ (Figure 2i,j; Figure S8, Supporting Information). The global stiffness ***G*** on the load direction was defined as the ratio of compression loading and deformation.

### Local Structural Parameters and Mechanical Property

1) Porosity. The micro structure of walnut shell cuboid samples (1mm × 1mm × *t*, *t* is walnut shell thickness; *n* = 10) were observed based on Micro‐CT images (Resolution 0.56 µm, Voltage 37KV, Skyscan1172, Bruker, Belgium) (Figure 3a). The cuboid zone (500 µm × 500 µm × *t*/20) were also selected and reconstructed every *t*/20 along thickness, the porosity of each cuboid zone was computed by CTAn (Bruker, Belgium). Then the variation rule of porosity in thickness direction can be obtained by fitting (Figure 3b). 2) Local elastic modulus. Three Micro‐CT images datasets from different walnut shell were employed to obtain the elastic modulus distribution along thickness. Eleven sections from outer to inner were divided on each Micro‐CT image dataset (Figure 3a). The cuboid zone (100µm × 100µm × 200 µm) in every section was selected and developed by Mimics 21.0 (Materialise Inc, Belgium). FE models were processed in Hypermesh 14.0 (Altair Inc, US) and Abaqus 2019 (Dassault Systems, US). The elastic modulus of different sections was obtained by simulation of static compression, which will be used to be describe the variation of modulus with the location of shell from outer to inner (Figure 3b). 3) Theoretical analysis, simulation and experiment. To study the effect of distribution of elastic modulus on walnut shell's mechanical properties, the theoretical analysis and Finite element analysis (FEA) were employed. The relationship of three elastic modulus (S‐shape gradient elastic modulus *E_SG_* (*i*); linear gradient distribution *E_L_* (*i*); uniform distribution *E_U_* (*i*)) and their position parameters were described. The S‐shape gradient distribution adopted the functional form to fit the relationship between the local elastic modulus and relative position of walnut shells as shown Figure 3b. The linear gradient distribution was obtained by linear interpolation of the maximum and minimum values of the S‐shape gradient distribution. Uniform distribution was the mean value of the S‐shape gradient distribution of elastic modulus in all ranges. The three distributions were normalized to avoid the influence of the base material (Figure 3h). Considering the symmetry of the structure, walnut shell was simplified to a 2D spherical shell whose thickness t was smaller than 1/10 of radius R (Figure 3i). Assuming small deformation, that is, contact impact only affects a small area of the shell; ignoring the shell cavity, the shell as a continuous medium; assuming that the shell material is orthotropic, that is, the mechanical properties of the same layer in the same plane, change along the direction of thickness. Variation rule of local elastic modulus of 2D models along thickness adopted the three elastic modulus distributions (S‐shape gradient, linear gradient, uniform distribution), respectively. The hertz elastic contact theory,^[^ ^12^ ^]^ was used to analyze the effect of elastic modulus distribution on the impact protection performance of walnut. 4) Spheric shell (radius is 10 mm and thickness is 1 mm) FE models were developed. The variation rule of elastic modulus along thickness were set as S‐shape gradient, linear gradient and uniform distribution according Equations (5)–(7), respectively (Figure S9, Supporting Information). The compressions on spheric shell were performed using numerical simulations (Abaqus 2019, Dassault Systems, US). The deflection and the stress/strain, the power was obtained when spheric shell was 500N force (Figure 3l; Figure S9, Supporting Information). 5) Three kinds of Samples (80mm × 20mm × 10 mm; *n* = 10) with uniform / linear gradient / S‐shape gradient porous microstructure (Figure 3m) was made by 3D printing (Form3, Formlab, USA) based on the porosity distribution forms of walnut shell along thickness. Gradient variation of porosity results in gradient variation of local elastic modulus (Figure 3C, Equation (3)). Three‐point bending testing of samples were processed and observed using universal testing machine (E10000, Instron, USA) and high‐speed cameras (i‐speed 3, ix‐cameras, UK) as shown in Figure 3m.

### Microstructure and Mechanical Analysis

1) Fracture surface observation. The fracture surface from outer to inner shell was observed using scanning electronic microscope (SEM, EVO MA15, ZEISS, Germany) as Figure 4f,g,i. Then the pit numbers in unit area were counted. The diameter of the pit is 2.31 ± 0.45 µm, and the mean pit numbers in per square micron area is 0.039 ± 0.003. 2) Single sclereid morphology. Walnut shells were sliced to 200 µm; immersed in cell dissociation buffer (1:1 for 70% acetic acid and 30% Hydrogen peroxide) 4 h at the temperature of 90 °C; 0.1% safranin O staining was processed for 5 min; scanned and observed by Confocal laser scanning microscope (Leica TCS SPE, Leica, Germany). Then morphology of intact single sclereids was obtained (Figure 4j). 3) Sclereid classification. Geometry models of sclereid of walnut shell were developed using Mimics (Mimics Medical 21.0, Materialise, Belgium) by Micro‐CT data as shown in Figure 4k (Video S4, Supporting Information). The volume and surface area of 210 sclereid models were calculated, then the surface area was compared with that of the tetrakaidekahedrons and cubes which had same volume. Intact sclereid were classified according to the quantity of sub‐cell (Figure 4l). 4) Fracture simulation of cellwall. A single walnut sclereid geometric model was developed by Micro‐CT data (Figure S10a, Supporting Information). Geomagic wrap 2017(3D systems, USA) was used to uniformly build pits on cellwall of sclereid (pit numbers in per square micron area is 0.039 ± 0.003). As a control, a sclereid model whose cellwall without pits was also built up. FE pre‐processing of the two models were performed in Hyperworks14.0 (Altair Inc, US), respectively. The inner surface of shell under compression was suffer 3D tensile stress which most likely induced the crack initiation (Figure S11, Supporting Information). Outward surface force was applied to the outer face of the two models, which simulated the loading condition of the sclereid at the crack tip. Simulations were performed in Abaqus 2019 (Dassault Systems, US). The micro crack propagation and growth on cellwall of the two models were compared (Figure 4h). 5) Deformation simulation of cell wall. Because of the unique shape of walnut shell sclereid, similar to the structure composed of many sub‐cells. It was found that the number of sclereid with 6 sub‐cells walnut shell is the most common (Figure 4l). Therefore, a 3D cubic cross model was established as the simplified model of sclereid l with 6 cell bodies. Elastic modulus is the same as walnut shell cellwall; the size of cubic cross model is 40 µm and its wall thickness is 1 µm. FE pre‐processing was performed in Hyperworks14.0 (Altair Inc, US), and simulations were performed in Abaqus 2019 (Dassault Systems, US). The displacement load is applied in one direction of model, which makes the model elongate in this direction, and the change of the relative cross‐section area and the relative volume of the inner cavity are calculated (Figure 4m; Figure S10b, Supporting Information).

### Statistical Analysis

Data comparisons throughout the manuscript are presented as mean ± standard error of the mean and plotted using Origin 2021(OriginLab, USA). All data sets were analyzed using *t*‐test or analysis of variance (ANOVA) with Origin 2021(OriginLab, USA). In cases whereby, samples do not meet normality criteria, a nonparametric test was used. A *p*‐value of less than 0.05 was considered statistically significant (* indicates *p* < 0.05, ** indicates *p* < 0.01, n.s. indicates no significant).

## Conflict of Interest

The authors declare no conflict of interest.

## Author Contributions

L.W. and P.X. contributed equally to this work. Conceptualization: L.Z.W., Y.B.F.; Methodology: L.Z.W., P.X., H.Y., Y.X.Y., W.K., J.L.L., Y.B.F.; Investigation: Y.B.F.; Visualization: L.Z.W., P.X.; Funding acquisition: L.Z.W., Y.B.F.; Project administration: L.Z.W., Y.B.F.; Supervision: Y.B.F.; Writing – original draft: L.Z.W., P.X., H.Y., Y.X.Y., W.K., J.L.L., Y.B.F.; Writing – review & editing: L.Z.W., P.X., Y.B.F.

## Supporting information

Supporting Information

Supplemental Video 1

Supplemental Video 2

Supplemental Video 3

Supplemental Video 4

## Data Availability

The data that support the findings of this study are available from the corresponding author upon reasonable request.
