# Supplementary material for: Fracture Resistance Biomechanisms of Walnut Shell with High‐Strength and Toughening
Source: Adv Sci (Weinh). 2023 Jul 30;10(27):2303238. doi: 10.1002/advs.202303238 (PMC10520628; doi:10.1002/advs.202303238)
Supplement: Supplementary file 1 — Supporting Information [file ADVS-10-2303238-s005.pdf]

ADVANCED SCIENCE
Open Access

Supporting Information

for *Adv. Sci.*, DOI 10.1002/advs.202303238

Fracture Resistance Biomechanisms of Walnut Shell with High-Strength and Toughening

*Lizhen Wang, Peng Xu, Huan Yin, Yanxian Yue, Wei Kang, Jinglong Liu and Yubo Fan**

Supporting Information

Fracture resistance biomechanisms of walnut shell with high-strength and toughening

*Lizhen.Wang[1]†, Peng.Xu[1]†, Huan.Yin[1], Yanxian. Yue[1], Wei. Kang[1], Jinglong. Liu[1], Yubo. Fan[1]**

[1]Key Laboratory of Biomechanics and Mechanobiology (Beihang University), Ministry of Education, Beijing Advanced Innovation Center for Biomedical Engineering, School of Biological Science and Medical Engineering, School of Engineering Medicine, Beihang University, Beijing, 100083, China.

†These authors contributed equally to this work: Lizhen Wang, Peng Xu.

*Corresponding author: Yubo Fan   Email: yubofan@buaa.edu.cn

SI Contents

Figures | S1-S12

Table | S1

Videos | S1-S4

Figures

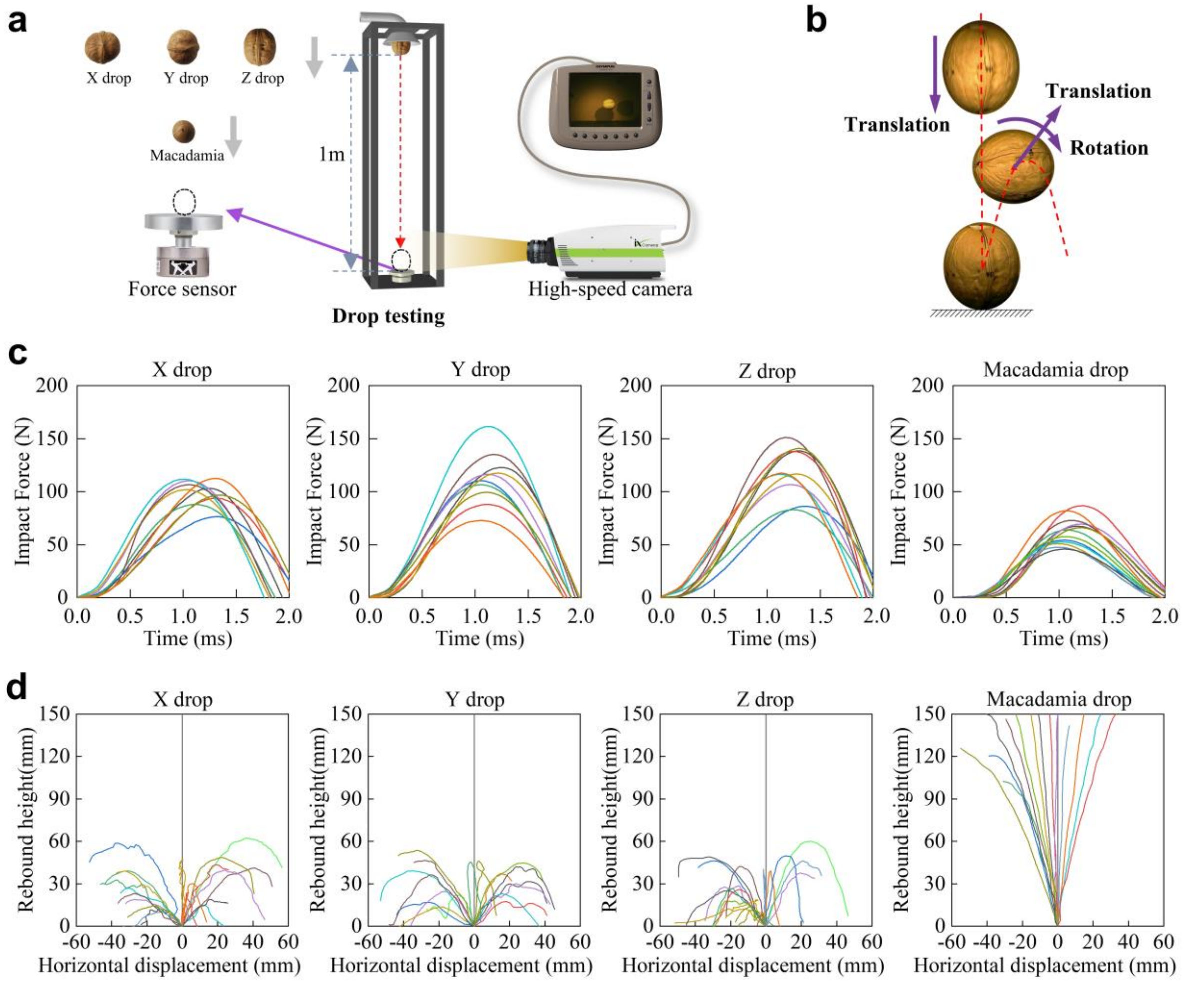

Supplementary Figure S1. Experimental equipment and impact force of drop testing

a. The equipment used to record drop track and impact force during nuts drop testing; **b.** Schematic of walnut's movement in dropping; **c.** the impact force of walnut and macadamia; the impact force of walnuts lager than macadamias during drop testing, obviously. This is because walnuts had greater mass. As shown in Figure 1c, when the peak accelerations were compared (the effort brought by the individual mass was eliminated), walnuts have better cushioning ability. **d.** the rebound track of walnuts and macadamia.

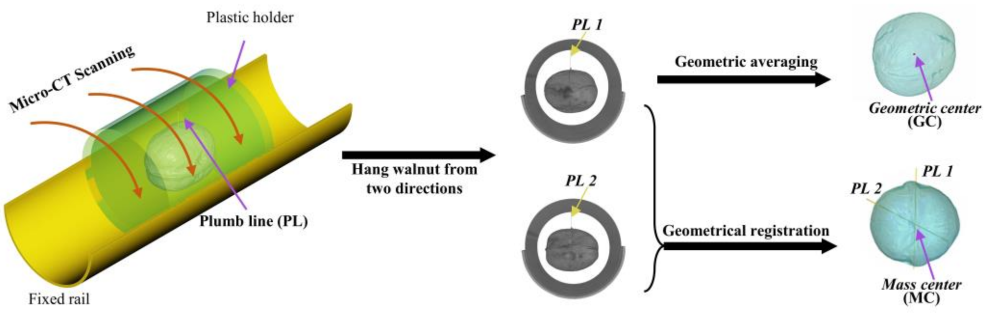

Supplementary Figure S2. lumb line method and Geometric reconstruction base on Micro-CT data were used to measure mass center and geometric center of walnut

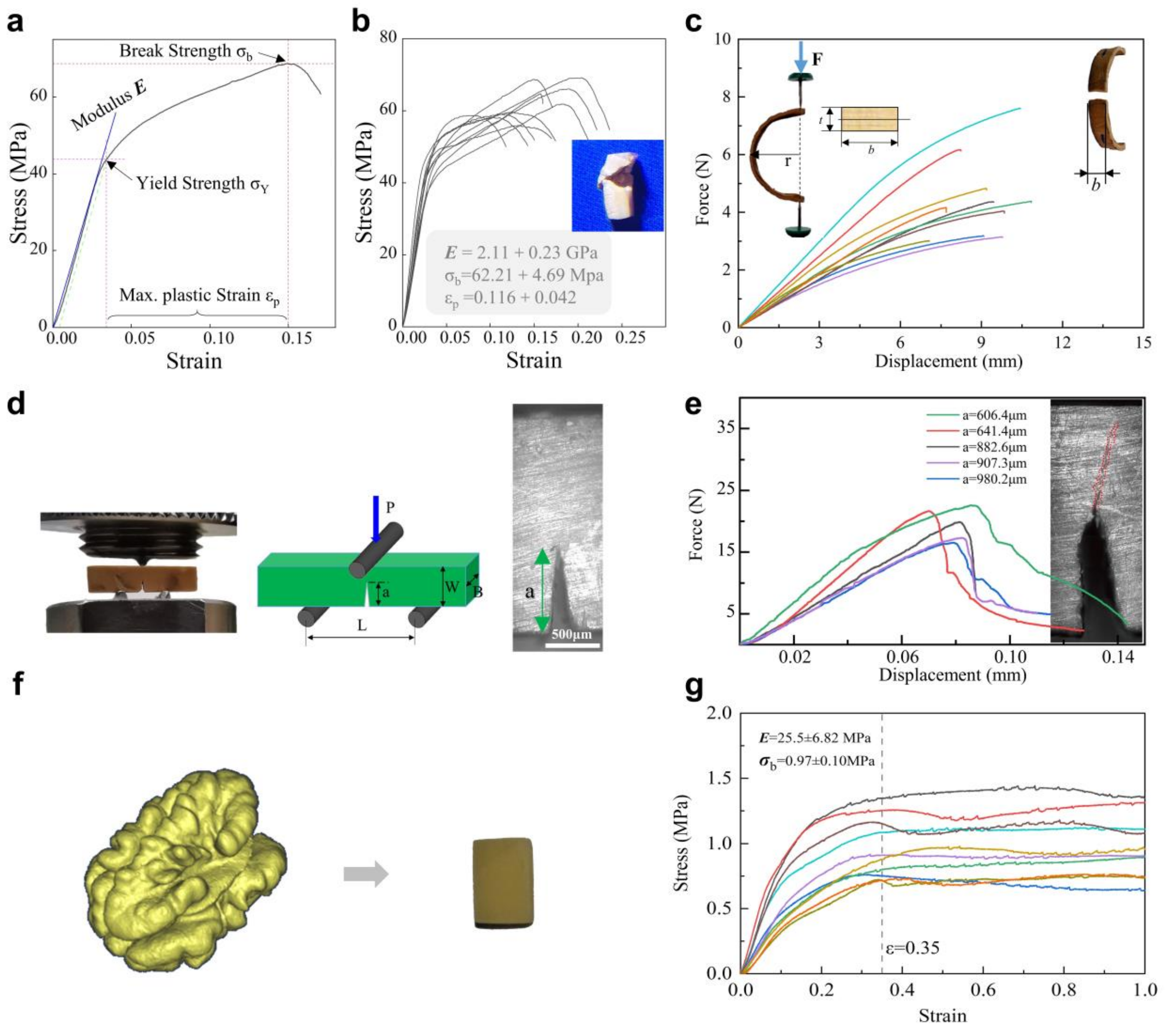

Supplementary Figure S3. Material properties testing results of walnut

a. Schematic diagram of material properties obtained from stress-strain curve of walnut compression testing; **b.** stress-strain curve of walnut shell from compression testing; **c.** load-displacement curve from C-ring testing; **d.** the geometry shape and size of three-points bending samples for toughness testing, "a" is the depth of pre-crack, "B" is the thickness of sample, "W" is the width of sample, and "L" is the span. For three-points bending used for toughness testing, the initial re-crack depth a should be about 0.5-0.75 of the width W of the sample, the ratio of thickness B to (W-a) should be 2-2.5, and the ratio of span to width L/W of the three-points bending sample should be about 4; **e.** load-displacement curve with different depth of pre-crack and the image of crack growth after toughness testing; f**.** the geometry shape of walnut kernel compression testing sample; **g.** Stress-strain curve from compression testing of walnut kernel. Stress-strain curve shows that the plastic deformation of kernel is very large. Zero slope in the curve was used as the basis for judging the yield point. The constitutive model of walnut kernel approximates the ideal elastic-plastic material model. ε=0.35 can be used as a criterion for kernel damage.

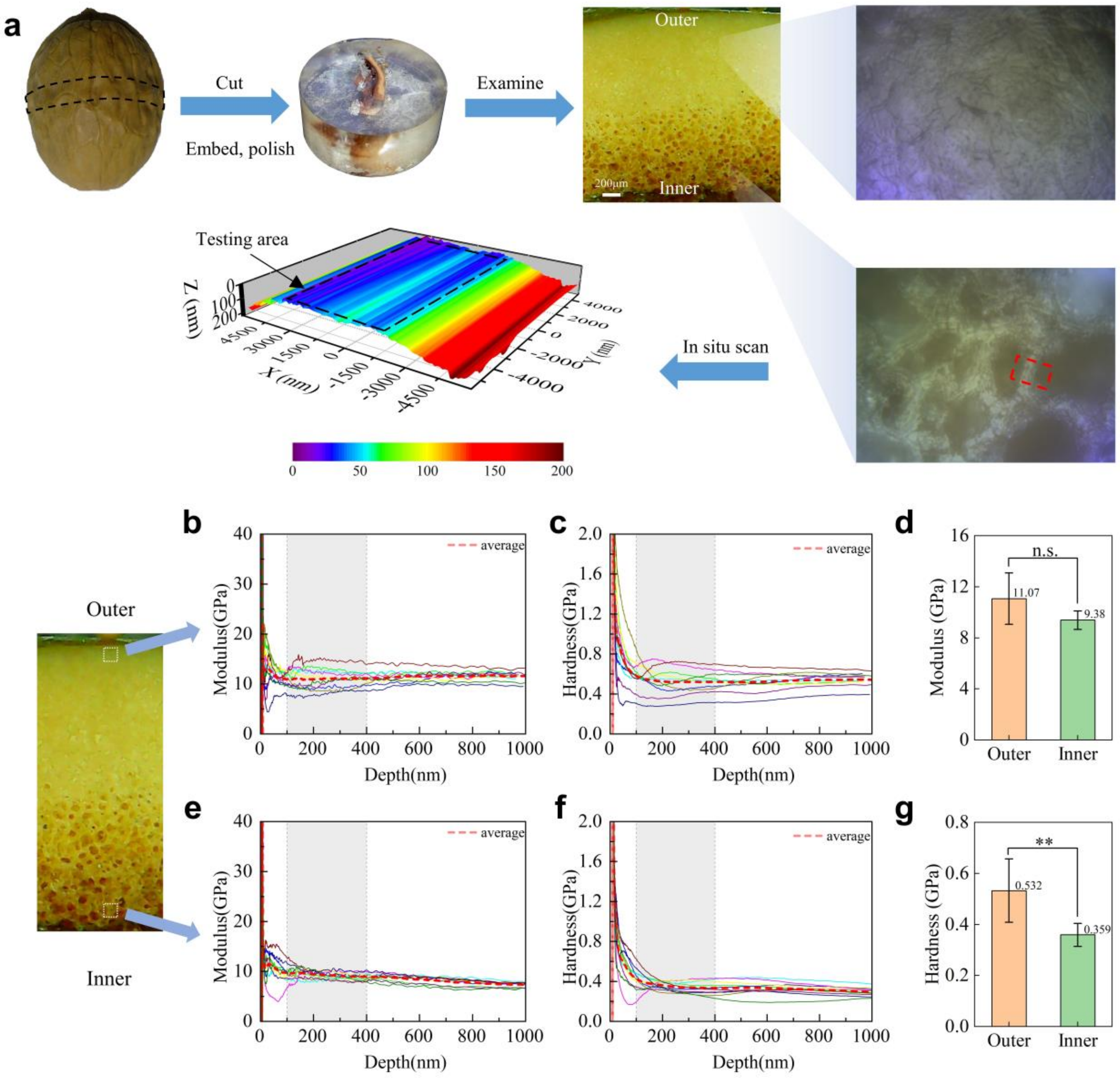

Supplementary Figure S4. Nanoindentation on the cellwall of walnut shell

a. the procedure of nanoindentation on the cellwall of walnut shell; **b** and **e** were the relationship between the cellwall modulus and the indent depth near the outer and inner aera of walnut shell, respectively; **c** and **f** were the relationship between the hardness of the cellwall and the indent depth near outer and inner aera the walnut shell, respectively; **d** and **g** show the statistical difference (*t*-tests) of cellwall modulus and hardness between outer and inner aera.

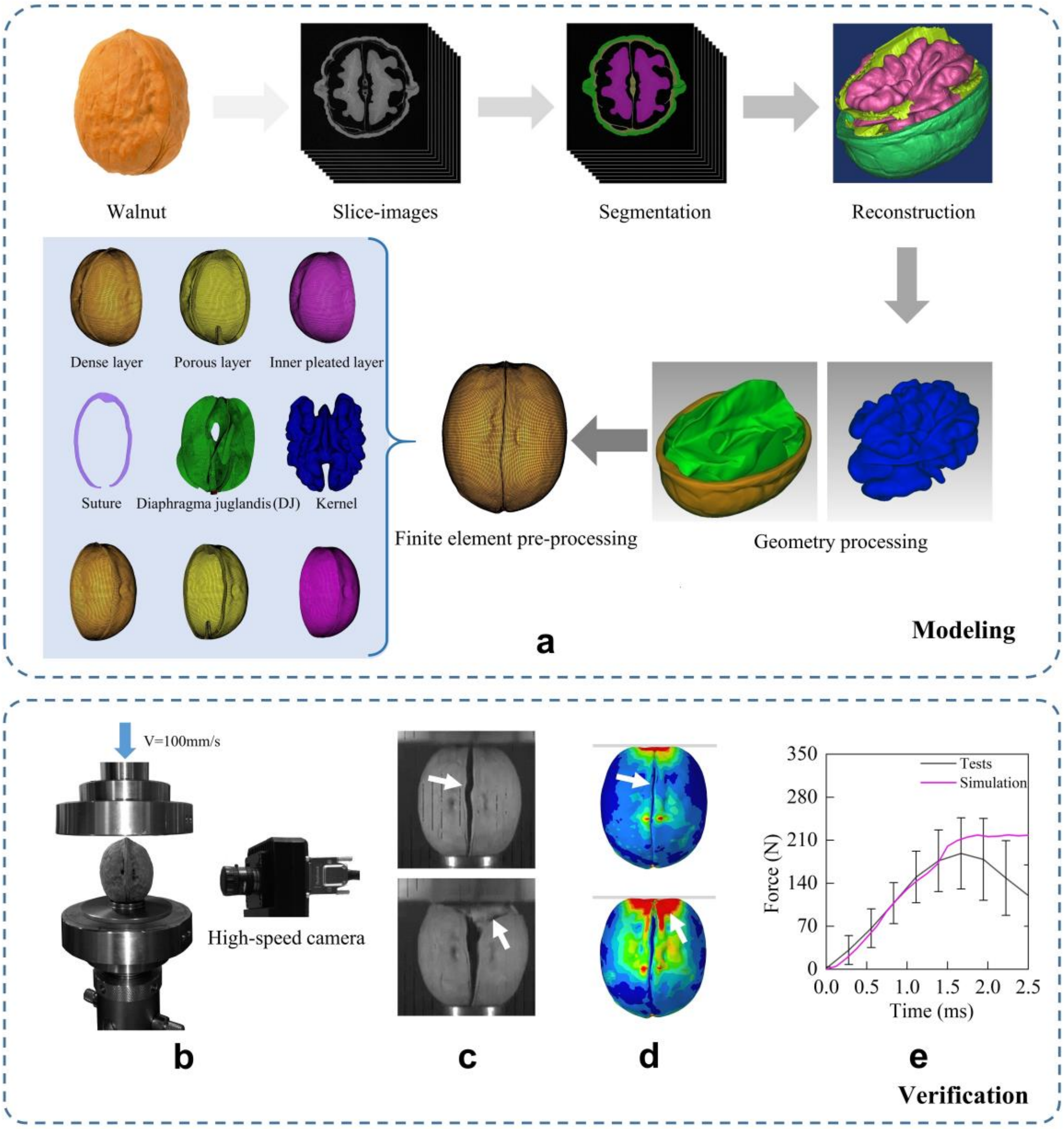

Supplementary Figure S5. Procedure of whole walnut finite element modeling and verification

a. The whole process from Micro-CT data to FE model; **b.** high speed compression (v=100mm/s) was used to verify the validity of walnut FE model; **c** and **d** shows the similar crack position and high-stress area between simulations and experiments; **e.** shows the impact forces were also very similar between simulations and experiments.

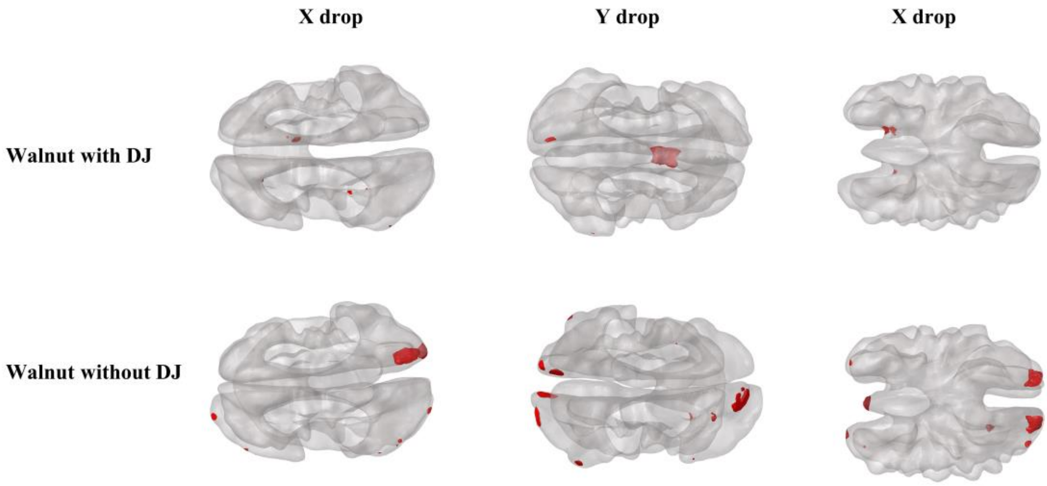

Supplementary Figure S6. kernel's damaged volume obtained by simulations of walnut drop

During the simulation, 0.35 strain was set as damage criterion of kernel. This figure shows the damage areas directly, it was supplement to Figure 2g and Figure 2h. Compared with walnut with DJ there are larger damaged volume in walnut without DJ no matter the drop direction.

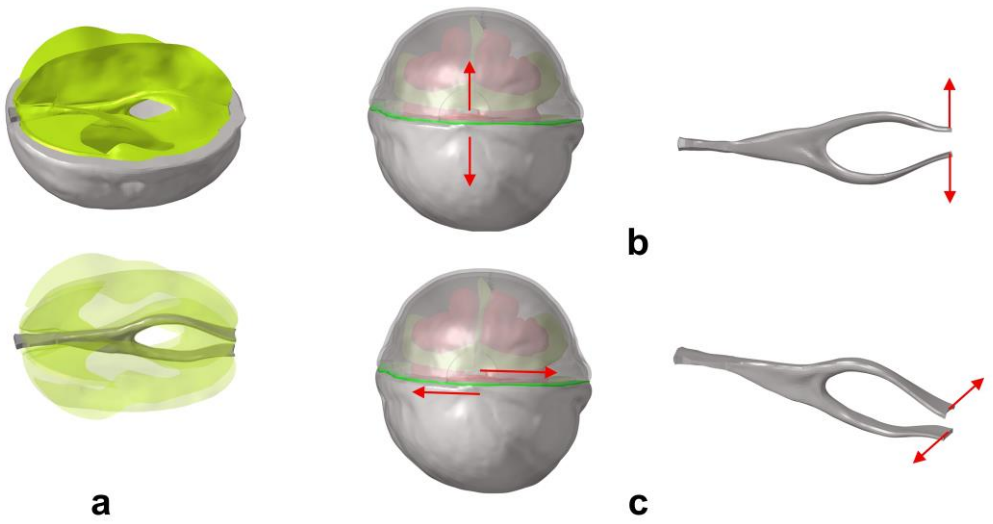

Supplementary Figure S7. Schematic diagram of the principle of DJ to prevent cracking of walnut

a. the structure of DJ; **b.** When the walnut is subjected to a pair of outward tensile forces perpendicular to the sutures, nut shell tends to crack along the sutures, and the support structure of DJ is tensile open, it will give the nut shell an inward reaction force at this time, thus reducing or counteracting the outward tensile force perpendicular to the sutures, so as to reduce the risk of cracking of the walnut; **c.** When the walnut is subjected to a pair of outward tensile forces parallel to the sutures, nut shell tends to crack along the sutures, and the support structure of DJ is twisted it will give the shell an inward reaction force at this time, thus reducing or counteracting the outward tensile force parallel to the sutures, so as to reduce the risk of cracking of the walnut.

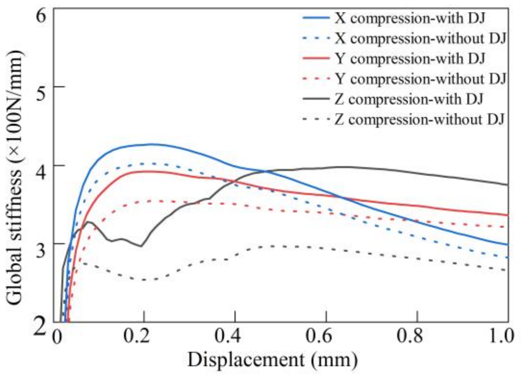

Supplementary Figure S8. Global stiffness of walnut shell varies with compression displacement

Compared with walnut without DJ, there are larger global stiffness of walnut with DJ no matter the drop direction.

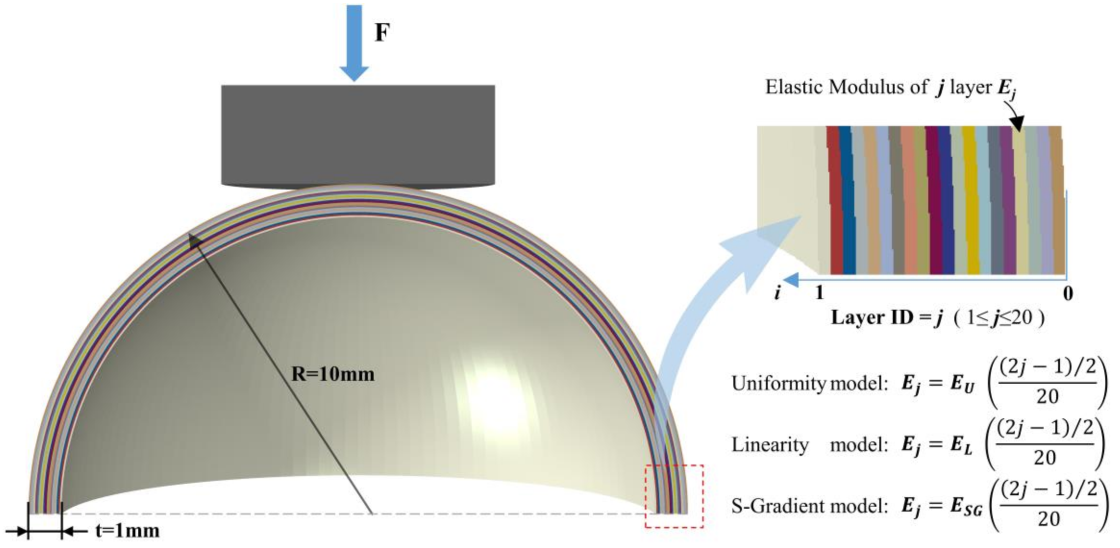

Supplementary Figure S9. Finite element model of gradient spherical shell under impact load

There are 20 layers in the spherical shell model. ***j*** is the layer ID form outer to inner. The elastic modulus of each layer was set based on the position on the *i* axis and elastic modulus distribution function (i.e., Equations (5)-(7), Figure 3h). The three FE models (S-shape gradient, linear gradient and uniform) use the same mesh element with different material properties. The boundary conditions and loading conditions of the three models are also consistent, and the lower end of the spherical shell is symmetrically constrained. All simulated the use of a rigid block to the spherical shell to exert a force of 500N, the rigid block acting on the spherical shell of the symmetric center.

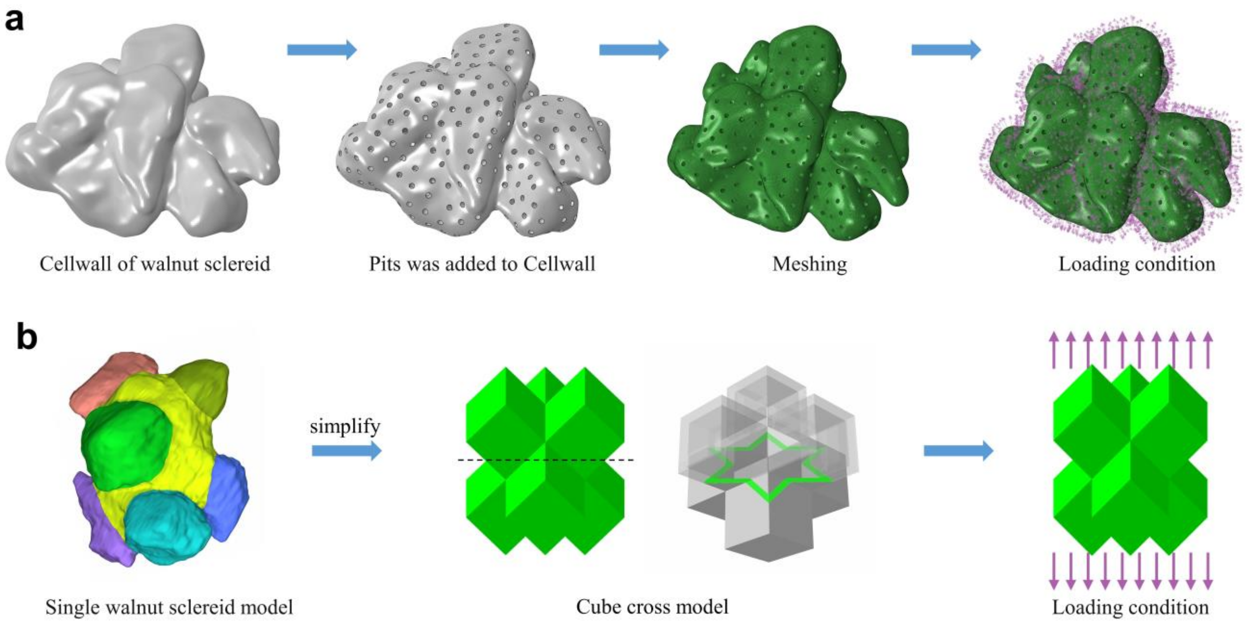

Supplementary Figure S10. Procedure of establishing walnut cell FE model.

a. Establishing single sclereid FE model for the analysis of cellwall fracture behavior: the geometric model of sclereid was built based on Micro-CT data; pits was uniformly added to cellwall; meshing and apply load of outward surface force on the outside of sclereid model. **b.** Establishing simplified cube cross model for the analysis of cellwall deformation: sclereid with 6 cell bodies was simplified as a three-dimensional cubic cross model; the elastic modulus is the same as walnut shell cellwall; the size of cubic cross model is 40μm and its wall thickness is 1μm; displacement load was applied in one direction of model, which makes the model elongate in this direction, and the change of the relative cross-section area and the relative volume of the inner cavity are calculated.

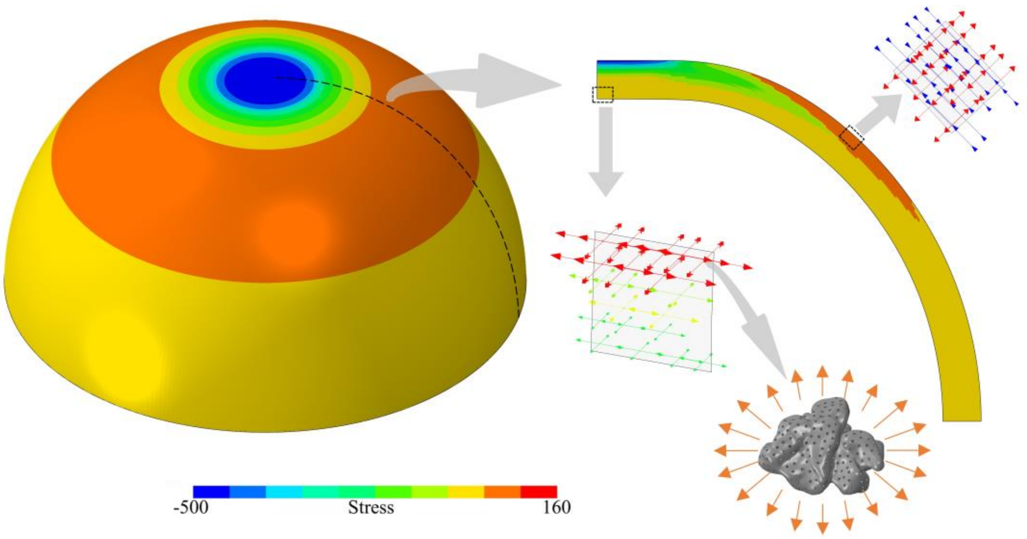

Supplementary Figure S11. Stress state of sclereid when walnut shell under compression

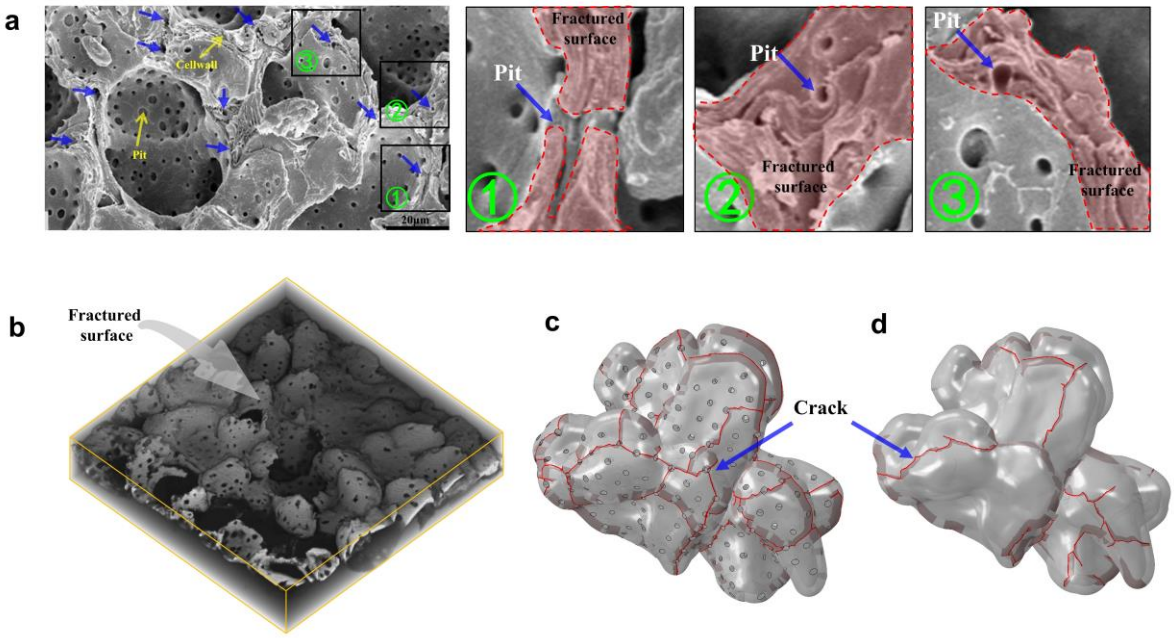

Supplementary Figure S12. Cracks on walnut shell sclereid

a. SEM image of the fracture interface of walnut shell; **b**. 3D topography of walnut shell fracture interface reconstructed from laser confocal microscope images; **c** and **d**, Crack propagation in the cell wall from simulation

Tables

Part	Mesh type	Element size (mm)	Section type	Thickness (mm)	Element type (LS-DYNA)	Material type (LS-DYNA)	Material properties (LS-DYNA) (g/mm^3, MPa)
Outer layer	Hexahedron+ Tetrahedron	0.4	solid	/	ELFORM=2	MATL3	Rho=0.001203, E=8700, NU=0.21, SIGY=48
Inner layer	Hexahedron+ Tetrahedron	0.4	solid	/	ELFORM=2	MATL3	Rho= 0.00027, E=1900, NU=0.21, SIGY=48
Inner pleated layer	Hexahedron	0.4	solid	0.2	ELFORM=2	MATL1	Rho= 0.00027, E=1700, NU=0.3
Diaphragma juglandis	Hexahedron+ Tetrahedron	0.4	solid	/	ELFORM=2	MATL1+ ADD_EROSION	Rho= 0.00027, E=1700, NU=0.3, SIGP1=40
Kernel	Tetrahedron	0.5	solid	/	ELFORM=2	MATL3	Rho= 0.001018, E=26, NU=0.4, SIGY=1, ETAN=0
Seed coat	Triangular	0.5	shell	0.18	ELFORM=5	MATL1	Rho= 0.00027, E=1700, NU=0.3
Suture	Hexahedron	0.25	solid	0.2	ELFORM=2	MATL1+ ADD_EROSION	Rho= 0.00027, E=1900, NU=0.21, SIGP1=2.5

Supplementary Table S1. Summary of whole walnut finite element model parameters

Videos

Supplementary Video 1.
Drop testing of walnut and macadamia based on high-speed camera and force transducer

Supplementary Video 2.
The anatomy structure and model of walnut

Supplementary Video 3.
Crack propagation of walnut shell samples after fracture

Supplementary Video 4.
The structure of walnut shell's single cell
